# Safety and efficacy of ultrasonic dissection versus electrocautery dissection in laparoscopic cholecystectomy for acute cholecystitis: an updated systematic review and meta-analysis

**DOI:** 10.1007/s00464-025-12132-2

**Published:** 2025-09-02

**Authors:** Ibrahim Moqbel, Mahmoud Albashier, Mario Maged Tawadros, Mohamed Basyouni Helal, Ahmed Hussein Abdelbaset, Ahmed Ihab Abdelmeguid, Razan Sulieman Abdalqader, Mohamed Youssef Abdou Youssef, Ashraf Abdelmonem Elsayed

**Affiliations:** 1 Faculty of Medicine, Cairo University, Cairo, Egypt; 2 Faculty of Medicine, Damietta University, New Damietta, Egypt; 3 Faculty of Medicine, Menoufia University, Menoufia, Egypt; 4 Al-Azher Faculty of Medicine, New Damietta University, New Damietta, Egypt; 5 Faculty of Medicine, Hashemite University, Zarqa, Jordan; 6 Faculty of Medicine, Zagazig University, Zagazig, Egypt; 7 General Surgery Department, Faculty of Medicine, Zagazig University, Zagazig, Egypt

**Keywords:** Laparoscopic cholecystectomy, Acute cholecystitis, Ultrasonic dissection, Electrocautery dissection

## Abstract

**Background:**

Laparoscopic cholecystectomy (LC) is the current gold standard for the management of acute cholecystitis, where ultrasonic dissection and electrocautery dissection are the two primary techniques used, however, it remains uncertain which approach is safer and more effective.

**Objectives:**

This systematic review and meta-analysis aimed to compare the clinical outcomes of ultrasonic dissection versus electrocautery dissection in LC for acute cholecystitis, particularly in operative time, hospital stay, blood loss, intraoperative and postoperative complications.

**Method:**

A systematic literature search was conducted in PubMed (MEDLINE), Scopus, Web of Science (WOS), EMBASE, and Cochrane CENTRAL up to January 7, 2025, following PRISMA guidelines. Eligible studies were randomized controlled trials (RCTs) comparing ultrasonic dissection and electrocautery dissection in LC for acute cholecystitis. Data extraction and risk-of-bias assessment were independently performed. Statistical analyses were conducted using Review Manager (RevMan 5.4), with heterogeneity assessed using I^2^ statistics and a random-effects model where necessary.

**Results:**

A total of 28 RCTs, comprising 3383 patients (1720 in the ultrasonic dissection group and 1663 in the electrocautery group), were included. The meta-analysis demonstrated that ultrasonic dissection significantly reduced operative time (− 9.13 min, 95% CI − 9.65 to − 8.61, *p* < 0.0001, I^2^ = 97%), hospital stay (− 0.95 days, 95% CI − 1.74 to − 0.17, *p* = 0.02, I^2^ = 100%), and blood loss (− 27.60 ml, 95% CI − 38.48 to − 16.72, *p* < 0.00001, I^2^ = 98%). Subgroup analysis reduced heterogeneity in some outcomes. Risk-of-bias assessment showed variability in study quality, with concerns in allocation concealment and performance bias in certain RCTs.

**Conclusion:**

Ultrasonic dissection demonstrated advantages over electrocautery dissection for laparoscopic cholecystectomy in patients with acute cholecystitis, including reduced operative time, lower blood loss, and shorter hospital stays. While some heterogeneity was observed among studies, ultrasonic dissection appears to be a safer and more efficient technique.

**Supplementary Information:**

The online version contains supplementary material available at 10.1007/s00464-025-12132-2.

Acute cholecystitis is a rapid-onset inflammation of the gallbladder, occurs in a matter of hours typically due to obstruction of the cystic duct or decreased blood flow (ischemia) of the gallbladder.

The worldwide prevalence of acute cholecystitis is nearly 10–15% [1], and the incidence has been rising gradually over the decades. Complications of cholecystitis include gangrenous cholecystitis, gallbladder perforation, and potentially life-threatening infections and prolonged hospitalizations that increase the economic burden of healthcare [2].

Laparoscopic cholecystectomy (LC) is the current gold standard for the management of acute cholecystitis [3]. It provides certain benefits compared to open surgery, such as faster recovery periods. Similar debate exists with ultrasonic dissection versus electrocautery dissection, each with its own benefits and risks [4], leading to disparate effects on operative time, blood loss, bile duct injury, and length of hospitalization.

Ultrasonic dissection uses high-frequency ultrasonic sound in cutting and coagulating tissue, offering less thermal spread, while electrocautery dissection uses high-energy electricity to cut or coagulate tissue, which could cause more thermal injury of surrounding tissue [5]. Despite the wide variety of studies and approaches, it remains uncertain which approach is the safest and most effective when performing LC for acute cholecystitis.

Therefore, this study is designed to compare the outcomes of patients with acute cholecystitis with either ultrasonic dissection or electrocautery to help clinical decision-making and improve patient outcomes for this common and relatively challenging condition.

## Methods

This study was conducted following the Cochrane Handbook for Systematic Reviews of Interventions [6]. The results were reported as specified by the Preferred Reporting Items for Systematic Reviews and Meta-Analyses (PRISMA) statement [7]. Additionally, this study has been registered with PROSPERO under the identification number (CRD420251020252).

### Data sources and search strategy

We systematically searched the PubMed (MEDLINE), Scopus, Web of Science (WOS), EMBASE, and Cochrane Central Register of Controlled Trials (CENTRAL) databases up to January 7, 2025. Tailored search terms and specific keywords were used for each database. The detailed search strategies of each database are presented in Supplementary Table 1.

### Study selection and eligibility criteria

This systematic review and meta-analysis was conducted in accordance with PRISMA guidelines. Eligible studies were randomized controlled trials (RCTs) comparing ultrasonic dissection with electrocautery dissection during laparoscopic cholecystectomy (LC) for acute cholecystitis, based on the following PICO criteria: Population—patients undergoing LC for acute cholecystitis; Intervention—ultrasonic dissection; Comparison—electrocautery dissection; and Outcomes—operative time, hospital stay, blood loss, lens cleaning, gallbladder perforation, intraoperative and postoperative complications, conversion to open surgery, bile leakage, postoperative collection, pain, nausea, stone spillage, readmission rate, and wound infection. Studies were excluded if they were non-human or in vitro, had duplicate or overlapping datasets, were non-original publications such as reviews, editorials, or guidelines, or were not published in English. Title and abstract screening was independently performed by three reviewers using Rayyan, followed by full-text assessment. Any discrepancies were resolved through discussion and consensus, with input from a senior author when necessary.

### Data extraction

Following the acquisition of full-text articles for all eligible studies, a pilot data extraction was conducted to refine and standardize the data collection process. A structured Microsoft Excel sheet was developed and organized into three main sections. The first section captured key study characteristics, including the study ID, country, total number of randomized patients, study duration (in months), setting, intervention, comparator, and the number of patients assigned to each group. The second section focused on the baseline characteristics of the participants, detailing the distribution of patients in both groups, age, sex, body mass index (BMI), and the rate of cholecystitis. The third section documented the predefined efficacy and safety outcome measures outlined in our protocol. Data extraction was independently performed by four reviewers to ensure consistency and accuracy. Any discrepancies encountered during the process were resolved through discussion, and when needed, a senior author was consulted to reach consensus.

### Risk of bias and certainty of evidence

The risk of bias was independently assessed by two reviewers using the Cochrane Risk of Bias 2 (RoB 2) tool [8]. Five key domains were evaluated: outcome measurement, selection of reported results, deviations from intended interventions, missing outcome data, and bias related to the randomization process. Any disagreements were resolved through discussion with a senior author. Two authors independently assessed the evidence's quality for indirectness, imprecision, inconsistency, and publication bias using the GRADE method. The evidence quality was further classified into four categories: very low, low, moderate, or high [46, 47]. After that, a summary table was created with GRADE profiler (version 3.6.1).

### Statistical analysis

All statistical analyses were conducted using Review Manager (RevMan) version 5.4. Continuous outcomes were evaluated using the mean difference (MD) with corresponding 95% confidence intervals (CI), while dichotomous outcomes were assessed using odds ratios (OR) and the Mantel–Haenszel method. Heterogeneity among the included studies was evaluated using both the Chi-square test and the I^2^ statistic. An I^2^ value exceeding 50% was interpreted as indicative of significant heterogeneity, warranting the use of a random-effects model. Heterogeneity was interpreted in accordance with the Cochrane Handbook (Chapter 9), with I^2^ values categorized as follows: 0–40% (low), 30–60% (moderate), 50–90% (substantial), and 75–100% (considerable). A Chi-square p-value of less than 0.1 was considered statistically significant for heterogeneity, while a p-value < 0.05 was deemed statistically significant for all other analyses.

Funnel plots were constructed to visually detect the risk of publication bias and any association between treatment estimated and sample size, in keeping with the recommendations by the Cochrane Collaboration, Egger’s regression test was performed using the standard error of the observed outcomes as predictors to detect asymmetry. Sensitivity analyses on clinically relevant outcomes were performed based on the qualitative evaluation of the studies included.

## Results

### Search and screening

**Fig. 1:**
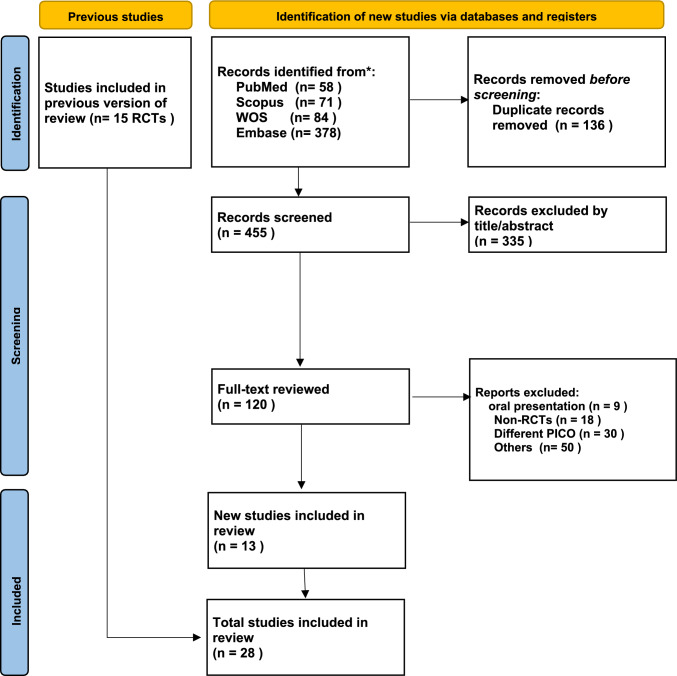
PRISMA 2020 flow diagram for updated systematic reviews which included searches of databases and registers only

As a result of the initial literature search, 591 articles were identified as potentially relevant. A total of 136 duplicates were removed, leaving 455 articles. A total of 335 were excluded due to unrelated research questions, study types, or participants. We thoroughly screened 120 full-text articles for eligibility. We found 28 relevant articles to include in our systematic review. The articles were all selected for a meta-analysis of the primary outcome [9–36] Fig. 1 shows the flow diagram of the search strategy and selection procedure (Fig. 1)

### Studies characteristics

In Table 1, the results of included studies are summarized. In total, 28 RCTs were included in the analysis. These studies included 3383 patients, of whom 1720 (50.84%) were assigned to ultrasonic dissection and 1663 (49.16%) to monopolar electrocautery. In the studies, the mean age of patients was between 20 and 70 years old, and among the patients, 32.45% were males. In Table 2, we summarize the main characteristics of the included studies.

**Table 1:** Patients characteristics of the included RCTs

No.	Study ID	Study arm	Sample size	Age	Male	BMI	Cholecystitis rate
1	Abdelhady et al. 2017	US	30	38.8 ± 12.7	8 (26.7%)	NA	NA
		Electrocautery	30	38.8 ± 12.7	6 (20%)		
2	Afzaal ahmed et al. 2019	US	72	34.5 ± 8.48	14 (19.4%)	NA	NA
		Electrocautery	72	36.25 ± 7.64	20 (27.8%)		
3	Akami et al. 2023	US	50	41.02*	16 (32%)	23.57*	NA
		Electrocautery	50	43.7*	11 (22%)	22.46*	
4	Baloch et al. 2015	US	43	43.62 ± 11.75	4 (10%)	NA	NA
		Electrocautery	43	44.19 ± 12	3 (7.3)		
5	Bessa et al. 2008	US	60	41.5 ± 10.3	13 (21.7%)	31.2 ± 3.50	NA
		Electrocautery	60	42.5 ± 11.4	12 (20%)	31 ± 40	
6	Bharti et al. 2023	US	50	40.30 ± 10.72	14 (28%)	NA	NA
		Electrocautery	50	38.72 ± 8.42	11 (22%)		
7	Blohm et al. 2024	US	152	59.68 ± 14.49	87 (57.2)	30 ± 8.5	12 (7.8%)
		Electrocautery	148	57.79 ± 14.55	68 (45.9)	29 ± 6.5	9 (6%)
8	Catena et al. 2014	US	21	71.6 ± 6.2	10 (10%)	28.1 ± 2.31	21 (100%)
		Electrocautery	21	71.2 ± 7.1	11 (11%)	26.6 ± 2.1	21 (100%)
9	Cengiz et al. 2005	US	43	46 (40, 47)	13 (30.2%)	27 ± 2.22	19 (44.1%)
		Electrocautery	37	44 (43, 50)	7 (18.9%)	27 ± 2.22	19 (51.3%)
10	Cengiz et al. 2010	US	73	45 ± 1.53	15 (20%)	27 ± 0.51	9 (12.3%)
		Electrocautery	79	47 ± 1.53	20 (25%)	27 ± 0.57	10 (12.6%)
11	Emad A. Ibrahim et al. 2022	US	60	49.44 ± 6.67	18 (30%)	26.98 ± 3.2	NA
		Electrocautery	60	48.62 ± 5.62	20 (33.33%)	27.52 ± 2.1	
12	Yehia et al. 2019	US	30	38.76 ± 13.1	6 (20%)	26.5 ± 1.8	NA
		Electrocautery	30	38.96 ± 13.03	8 (26.7%)	26.1 ± 2.1	
13	Jain et al. 2013	US	100	39.55 ± 11.12	6 (6%)	NA	NA
		Electrocautery	100	38.67 ± 11.87	11 (11%)		
14	Kandil et al. 2010	US	70	40.97 ± 11.56	29 (29%)	28.14 ± 3.87	NA
		Electrocautery	70	41.38 ± 11.91	30 (30%)	28.64 ± 4.46	
15	Liao et al. 2016	US	117	42.2 10.4	51 (51%)	24.6 ± 3.1	NA
		Electrocautery	81	43.4 11.1	51 (40%)	25.0 ± 3.6	
16	Mahabaleshwar et al. 2011	US	30	45.3*	8 (26.7%)	27.53*	NA
		Electrocautery	30	47.36*	12 (40%)	27.53*	
17	Manoj et al. 2023	US	150	46.53 ± 13.740	13 (8.70%)	NA	NA
		Electrocautery	150	45.3 ± 13.961	84 (56%)		
18	Mattila et al. 2015	US	88	47.0 ± 11	21 (33%)	27.2 ± 3.7	NA
		Electrocautery	79	45.0 ± 13	4 (25%)	27.6 ± 3.8	
19	Nadim Khan et al. 2012	US	64	37.28 ± 14.4	39 (19%)	NA	NA
		Electrocautery	64	35.98 ± 13.29	39 (20%)		
20	Nakeeb et al. 2010	US	60	NA	42 (70%)	NA	NA
		Electrocautery	60		35 (58.3)		
21	Prakash B et al. 2023	US	24	NA	13 (13%)	NA	NA
		Electrocautery	24		14 (14%)		
22	Redwan et al. 2010	US	80	34.56*	27 (27%)	NA	NA
		Electrocautery	80	40.88*	27 (33%)		
23	Shabbir et al. 2016	US	60	36.41 ± 6.24	16 (26.67%)	NA	NA
		Electrocautery	60	35.88 ± 6.52	19 (31.67%)		
24	Sista et al. 2014	US	22	57.2 ± 13	9 (9%)	NA	NA
		Electrocautery	21	58.1 ± 11	8 (8%)		
25	Tempe´ et al. 2013	US	40	43.2 ± 11.8	13 (13%)	27.1 ± 4.4	NA
		Electrocautery	33	45.8 ± 11.6	7 (7%)	26.2 ± 2.9	
26	Ulla et al. 2024	US	35	41.33 ± 11.77	12 (12%)	NA	NA
		Electrocautery	35	39.73 ± 7.43	10 (10%)		
27	Ramzanali et al. 2013	US	46	40 ± 7.84	7 (7%)	NA	NA
		Electrocautery	46	40 ± 7.84	8 (8%)		
28	Mathur et al. 2021	US	50	40.20 ± 10.79	12 (12%)	NA	NA
		Electrocautery	50	39.88 ± 8.54	20 (20%)		
29	Gelmini et al. 2010	US	95	52.05 ± 18.13	37 (38.95%)	22.6	NA
		Electrocautery	90	51.08 ± 16.41	37 (41.11%)	22.6	

* Report as mean

**Table 2:** Summary characteristics of RCTs included in the systematic review

	Study ID	Country	Total of randomized Pts	Duration of study (months)	Setting	Intervetion	Compartor	No. pts of intervention	No pts of compartor
1	Abdelhady et al. 2017	Egypt	60	24	Elective	US	Electrocautery	30	30
2	afzaal ahmed et al. 2019	Pakistan	144	14	Elective	US	Electrocautery	72	72
3	Akami et al. 2023	India	100	24	Elective	US	Electrocautery	50	50
4	Baloch et al. 2015	Pakistan	86	12	Elective	US	Electrocautery	43	43
5	Bessa et al. 2008	Egypt	120	12	Elective	US	Electrocautery	60	60
6	Bharti et al. 2023	India	100	12	Elective	US	Electrocautery	50	50
7	Blohm et al. 2024	Sweden	300	48	Elective	US	Electrocautery	152	148
8	Catena et al. 2014	Italy	42	24	Elective	US	Electrocautery	21	21
9	cengiz et al. 2005	Sweden	80	24	Elective	US	Electrocautery	43	37
10	Cengiz et al. 2010	Sweden	243	19	Elective	US	Electrocautery	73	81
11	Emad A. Ibrahim et al. 2022	Saudi Arabia	120	36	NR	US	Electrocautery	60	60
12	Yehia et al. 2019	Egypt	60	7	Elective	US	Electrocautery	30	30
13	Jain et al. 2013	India	200	6	Elective	US	Electrocautery	100	100
14	Kandil et al. 2010	Egypt	140	12	Elective	US	Electrocautery	70	70
15	Liao et al. 2016	China	234	32	Elective	US	Electrocautery	117	81
16	Mahabaleshwar et al. 2011	India	60	12	Elective	US	Electrocautery	30	30
17	Manoj et al. 2023	India	300	24	Elective	US	Electrocautery	150	150
18	Mattila et al. 2015	Finland	169	30	Elective	US	Electrocautery	88	79
19	Nadim Khan et al. 2012	Pakistan	128	6	Elective	US	Electrocautery	64	64
20	Nakeeb et al. 2010	Egypt	120	18	Elective	US	Electrocautery	60	60
21	Prakash B et al. 2023	India	48	2	Elective	US	Electrocautery	24	24
22	Redwan et al. 2010	Egypt	160	18	Elective	US	Electrocautery	80	80
23	Shabbir et al. 2016	Pakistan	120	12	Elective	US	Electrocautery	60	60
24	sista et al. 2014	Italy	43	84	Elective	US	Electrocautery	22	21
25	Tempe´ et al. 2013	Sweden	80	24	Elective	US	Electrocautery	40	33
26	Ulla et al. 2024	India	70	12	Elective	US	Electrocautery	35	35
27	Ramzanali et al. 2013	Pakistan	92	6	Elective	US	Electrocautery	46	46
28	Mathur et al. 2021	India	100	18	Elective	US	Electrocautery	50	50

Question: US compared to electurcauterey for laparoscopic cholecystectomy. Setting: Bibliography: US versus electurcauterey for laparoscopic cholecystectomy. Cochrane Database of Systematic Reviews

### Risk of bias and certainty of evidence

**Fig. 2:**
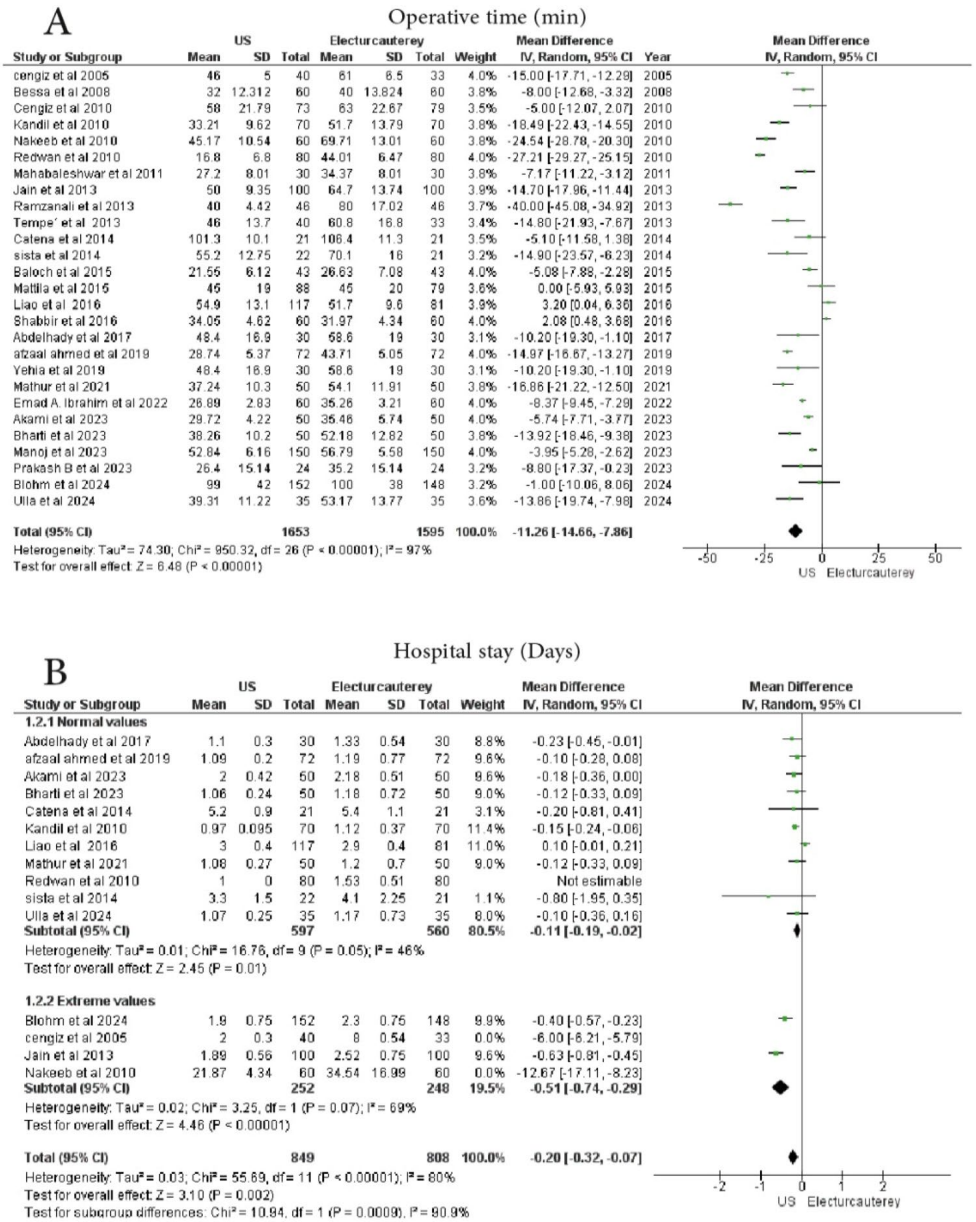
Forest plot show **A** operative time, **B** hospital stay

As shown in Fig. 2, the included studies' methodological quality was evaluated using the "Risk of Bias" assessment tool. In twenty RCTs, random sequence generation generated low bias and unclear bias in eight RCTs. One RCT showed a high risk of bias due to allocation concealment, 12 RCTs showed an unclear risk of bias, and fifteen RCTs showed an unclear risk of bias. A high-performance bias was found in one RCT, a low-performance bias in six RCTs, and an unclear performance bias in twenty-one RCTs. One RCT had a high detection bias, nine had a low detection bias, and 18 had an unclear detection bias. Six RCTs had a high risk of attrition bias, and twenty-two had a low risk. One RCT had low reporting bias, seven had high reporting bias, and the remaining RCTs were unclear (Supplementary File Fig. 1). According to GRADE, the certainty of evidence was high for gallbladder perforation (important); moderate for operative time and length of hospital stay (important); and low for postoperative complications, conversion to open, and bile leak (critical), as well as intraoperative (critical). Table 3 also the remaining of grade assessment to other outcomes is hold on (Supplementary File Table S3).

**Table 3:** Grading of recommendations assessment, development, and evaluation (GRADE) evidence profile

Certainty assessment							No. of patients		Effect		Certainty	Importance
№ of studies	Study design	Risk of bias	Inconsistency	Indirectness	Imprecision	Other considerations	US	Electurcauterey	Relative (95% CI)	Absolute (95% CI)		
*Operative time (min)*												
27	Randomised trials	Serious^a^	Very serious^b^	Not serious	Not serious	Very strong association	1653	1595	–	MD 11.26 lower (14.66 lower to 7.86 lower)	⨁⨁⨁◯ Moderate^a,b^	Important
*Hospital stay (days)*												
15	Randomised trials	Serious^a^	Very serious^b^	Not serious	Not serious	Very strong association	949	901	–	MD 0.95 lower (1.74 lower to 0.17 lower)	⨁⨁⨁◯ Moderate^a,b^	Important
*Gall bladder perforation*												
21	Randomised trials	Serious^a^	Not serious	Not serious	Not serious^c^	Very strong association	249/1357 (18.3%)	364/1350 (27.0%)	OR 0.45 (0.31 to 0.66)	127 fewer per 1000 (from 167 to 74 fewer)	⨁⨁⨁⨁ High^a,c^	Important
*Interoperative complication*												
9	Randomised trials	Serious^a^	Not serious	Not serious	Serious^d^	None	59/571 (10.3%)	76/561 (13.5%)	OR 0.72 (0.46 to 1.15)	34 fewer per 1000 (from 68 fewer to 17 more)	⨁⨁◯◯ Low^a,d^	Critical
*Postoperative complication*												
14	Randomised trials	Serious^a^	Not serious	Not serious	Serious^d^	None	70/909 (7.7%)	102/860 (11.9%)	OR 0.62 (0.41 to 0.93)	42 fewer per 1000 (from 66 to 7 fewer)	⨁⨁◯◯ Low^a,d^	Critical
*Conversion to open*												
14	Randomised trials	Serious^a^	Not serious	Not serious	Serious^d^	None	17/942 (1.8%)	33/895 (3.7%)	OR 0.53 (0.28 to 0.99)	17 fewer per 1000 (from 26 to 0 fewer)	⨁⨁◯◯ Low^a,d^	Critical
*Bile leak*												
20	Randomised trials	Serious^a^	Not serious	Not serious	Serious^d^	None	36/1206 (3.0%)	83/1150 (7.2%)	OR 0.40 (0.27 to 0.61)	42 fewer per 1000 (from 52 to 27 fewer)	⨁⨁◯◯ Low^a,d^	Critical

*CI* confidence interval, *MD* mean difference, *OR* odds ratio

^a^Some studies show unclear risk of bias

^b^Heterogenicity is very high

^c^Not downgraded for imprecision: precise effect (OR 0.31–0.66) with adequate information size

^d^CI includes important benefit and harm (OR 0.46–1.15) with insufficient information size

### Outcomes

A total of 28 studies were included in our quantitative analysis. The outcomes analyzed in this meta-analysis were: operative time, hospital stay, blood loss, lens cleaning, gallbladder perforation, intraoperative complications, postoperative complications, conversion to open surgery, bile leakage, postoperative collection, postoperative pain, postoperative nausea, CBD injury, stone spillage, readmission rates, and wound infections.

#### Operative time (minutes)

A meta-analysis of 27 studies assessed the operative time in the laparoscopic cholecystectomy group. The 27 studies reported the mean operating time with standard deviation. A total of 1653 patients were treated with laparoscopic cholecystectomy under US guidance, while 1595 patients underwent the procedure using electrocautery. There was a significant difference in heterogeneity among the studies, and the mean difference between the two groups was − 9.13 min (95% CI − 9.65 to − 8.61, *p* < 0.0001), with considerable heterogeneity (I^2^ = 97%). Figure 2A, Publication bias was assessed through a funnel plot shown at (Supplementary File Figure S2). The Egger's regression test’s P-value = 0.00149 indicates that there is no publication bias.

#### Hospital stay (days)

Fifteen studies reported the postoperative length of hospital stay with standard deviation. A total of 949 patients underwent laparoscopic cholecystectomy under US guidance, and 901 patients were treated with electrocautery. A statistically significant reduction in hospital stay was observed in the US guidance group (mean difference − 0.95 days, 95% CI − 1.74 to − 0.17, *p* = 0.02), with considerable heterogeneity (I^2^ = 100%).

In a subgroup analysis to reduce heterogeneity, 11 studies reported the postoperative length of hospital stay. A total of 597 patients underwent laparoscopic cholecystectomy under US guidance, and 560 patients received electrocautery. The results were statistically significant (*p* = 0.01), and moderate heterogeneity (I^2^ = 46%) was observed (MD = − 0.11, 95% CI − 0.19 to − 0.02).

Furthermore, 4 studies showed a statistically significant reduction in hospital stay with US guidance (*p* = 0.008), but significant heterogeneity (I^2^ = 100%) was present (MD = − 4.16, 95% CI − 7.25 to − 1.06) (Fig. 2B)

#### Blood loss (ml)

Ten studies analyzed blood loss between the two groups. A total of 729 patients received laparoscopic cholecystectomy under US guidance, and 686 patients underwent electrocautery. The electrocautery group demonstrated significantly higher blood loss (MD = − 27.60, 95% CI − 38.48 to − 16.72, *p* < 0.00001), with considerable heterogeneity (I^2^ = 98%).

**Fig. 3:**
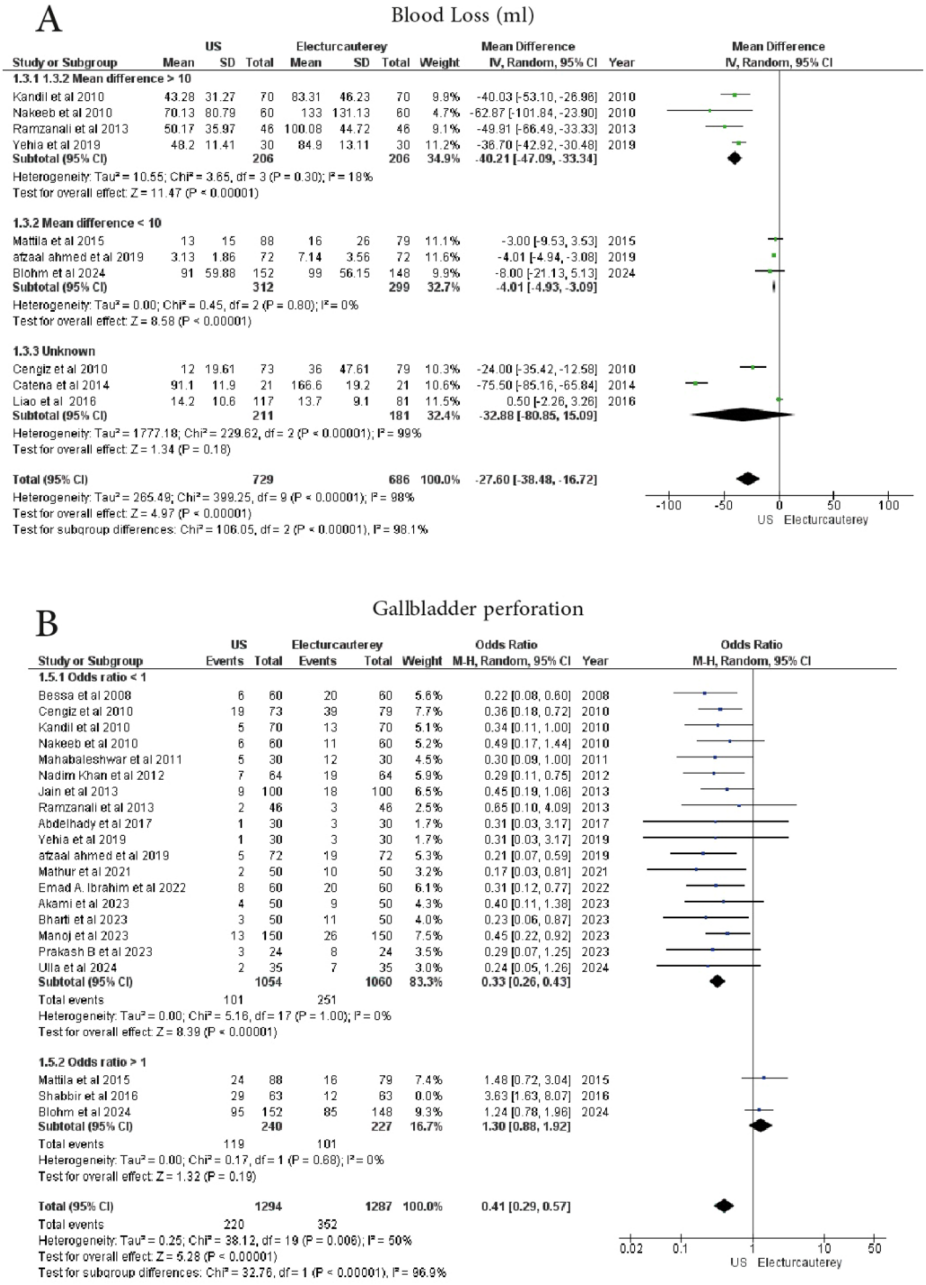
Forest plot show **A** blood loss, **B** gallbladder perforation

To reduce heterogeneity, a subgroup analysis of 4 studies was performed, which showed 206 patients in each group. The findings were statistically significant (*p* < 0.00001), with low heterogeneity (I^2^ = 18%) (MD = − 40.21, 95% CI − 47.09 to − 33.34). In another subgroup of 3 studies involving 312 patients in the US guidance group and 299 patients in the electrocautery group, a statistically significant result was found (*p* < 0.00001), with no heterogeneity (I^2^ = 0%) (MD = − 4.01, 95% CI − 4.93 to − 3.09). However, in a third subgroup of 3 studies, 211 patients in the US guidance group and 181 patients in the electrocautery group, the findings were statistically insignificant (*p* = 0.18), with considerable heterogeneity (I^2^ = 99%) (MD = − 32.88, 95% CI − 80.85 to − 15.09) (Fig. 3A)

#### Gallbladder perforation

A total of 21 studies reported on gallbladder perforation. 1357 patients underwent electrocautery and 1357 patients received laparoscopic cholecystectomy under US guidance. A significant reduction in gallbladder perforation was observed in the US guidance group (OR = 0.45, 95% CI 0.31–0.66, *p* = 0.0001), with substantial heterogeneity (I^2^ = 67%).

A subgroup analysis of 18 studies showed a statistically significant result (*p* = 0.00001) with no heterogeneity (I^2^ = 0%) (OR = 0.33, 95% CI 0.26–0.43). However, 3 studies with 290 patients undergoing electrocautery and 303 patients undergoing US-guided surgery showed statistically insignificant results (*p* = 0.07) with substantial heterogeneity (I^2^ = 62%) (OR = 1.76, 95% CI 0.96–3.25). However, by leaving on out, when excluding the study by Shabbir et al. (2016), the findings remained statistically significant (*p* < 0.00001), with moderate heterogeneity (I^2^ = 50%) (MD = 0.41, 95% CI 0.29–0.57) (Fig. 3B)

#### Bile leakage

**Fig. 4:**
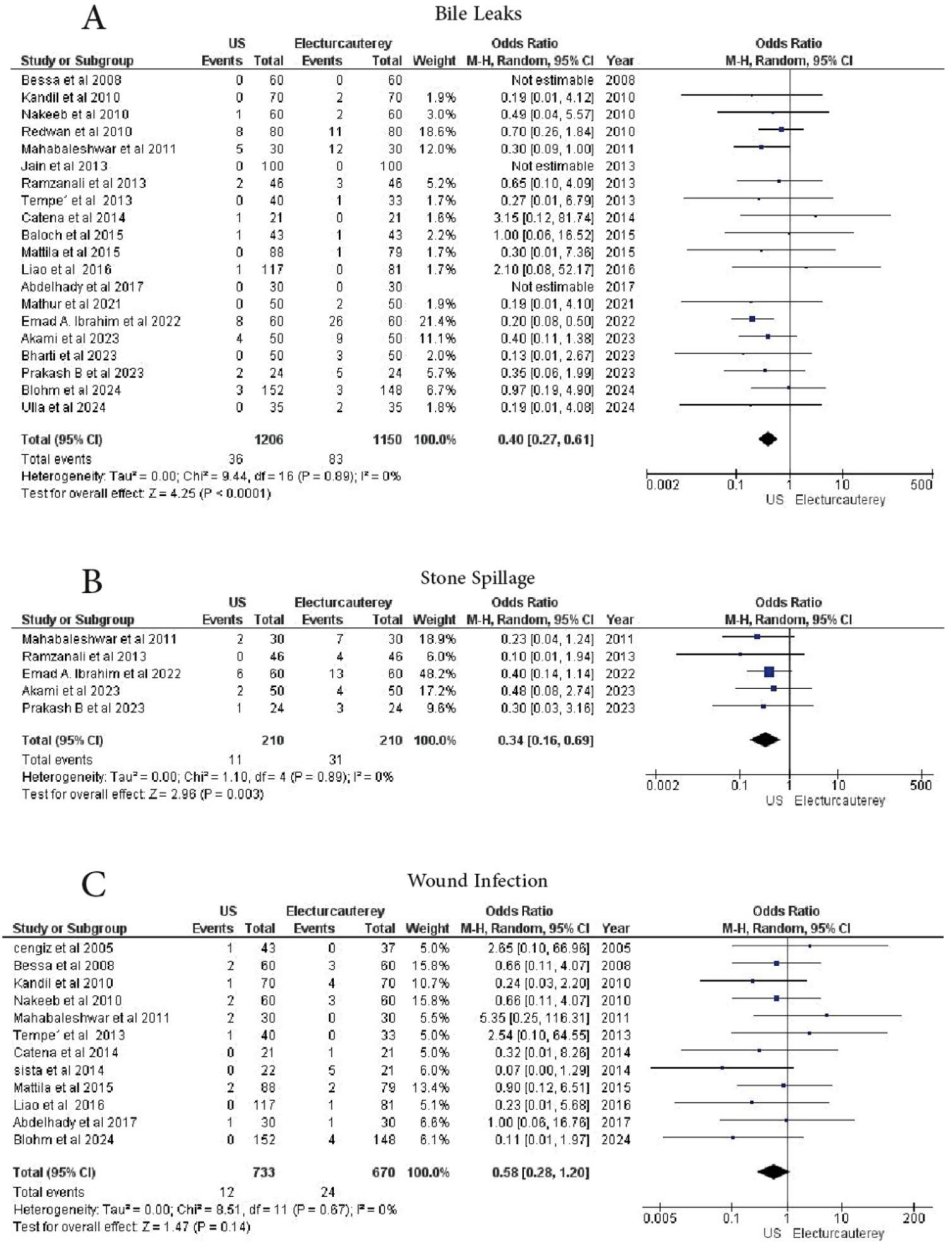
Forest plot show **A** bile leak, **B** stone sptinge, **C** wound infection

A total of 20 studies reported bile leakage. 1206 patients underwent laparoscopic cholecystectomy under US guidance, and 1150 patients received electrocautery. A significant reduction in bile leakage was observed in the US guidance group (OR = 0.40, 95% CI 0.27–0.61, *p* = 0.0001), with no heterogeneity (I^2^ = 0%) (Fig. 4A)

#### Stone spillage

5 studies reported on stone spillage. A total of 210 patients underwent laparoscopic cholecystectomy under US guidance, and 210 patients received electrocautery. A statistically significant reduction in stone spillage was observed in the US guidance group (OR = 0.34, 95% CI 0.16–0.69, *p* = 0.0003), with no heterogeneity (I^2^ = 0%) (Fig. 4B)

#### Wound infection

A total of 12 studies reported on wound infections. 733 patients underwent laparoscopic cholecystectomy under US guidance, and 670 patients were treated with electrocautery. No statistically significant difference was observed (OR = 0.58, 95% CI 0.28–1.20, *p* = 0.14), with no heterogeneity (I^2^ = 0%) (Fig. 4C)

#### Intraoperative complications

**Fig. 5:**
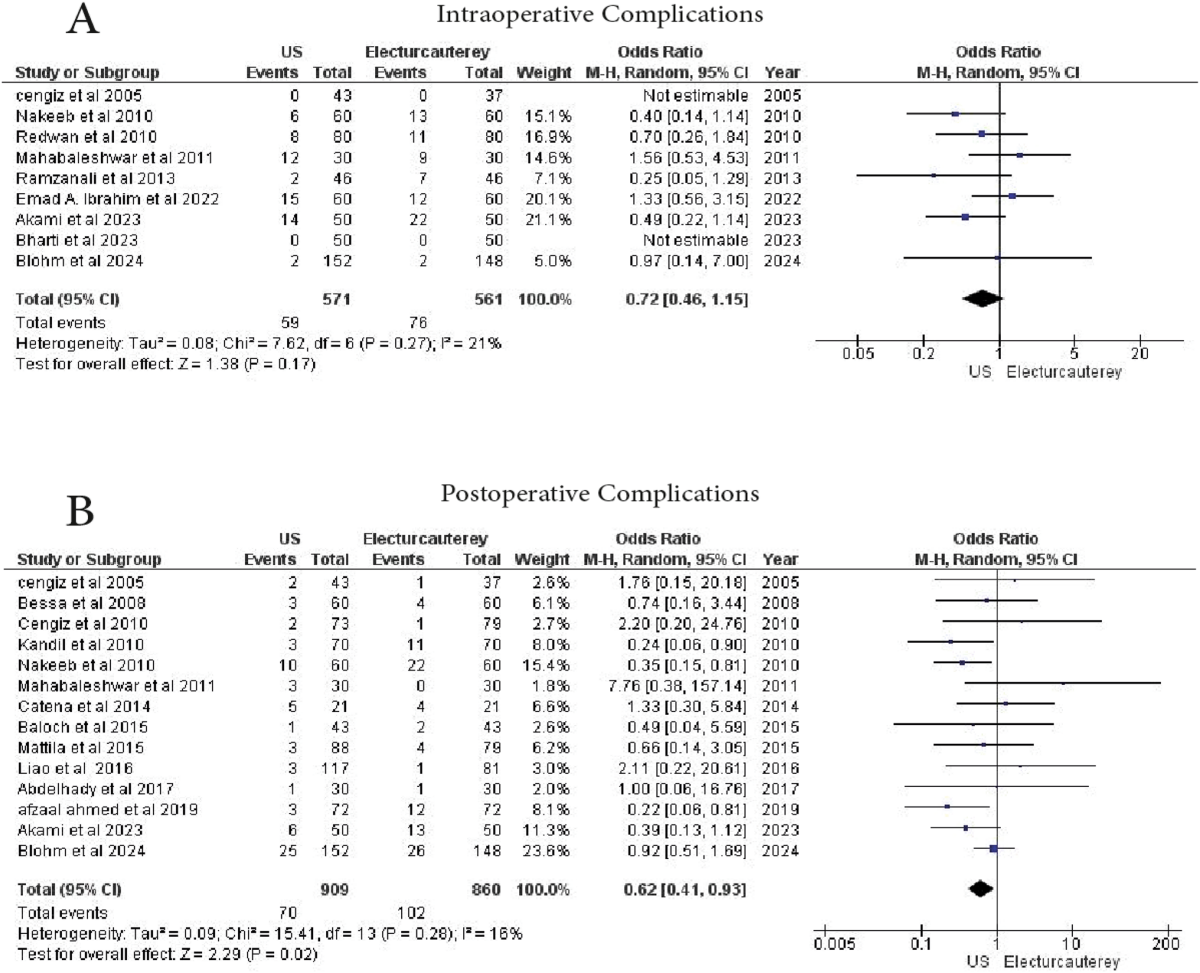
Forest plot show **A** intraoperative complication, **B** postoperative complications

A total of 9 studies reported intraoperative complications. 571 patients underwent laparoscopic cholecystectomy under US guidance, and 561 patients were treated with electrocautery. No statistically significant difference was found between the two groups (OR = 0.72, 95% CI 0.46–1.15, *p* = 0.17), with low heterogeneity (I^2^ = 21%) (Fig. 5A)

#### Postoperative complications

A total of 14 studies reported postoperative complications. 860 patients underwent electrocautery, and 909 patients underwent laparoscopic cholecystectomy under US guidance. The US guidance group showed a significant reduction in postoperative complications (OR = 0.62, 95% CI 0.41–0.93, *p* = 0.02), with low heterogeneity (I^2^ = 16%) (Fig. 5B)

#### Postoperative collection

**Fig. 6:**
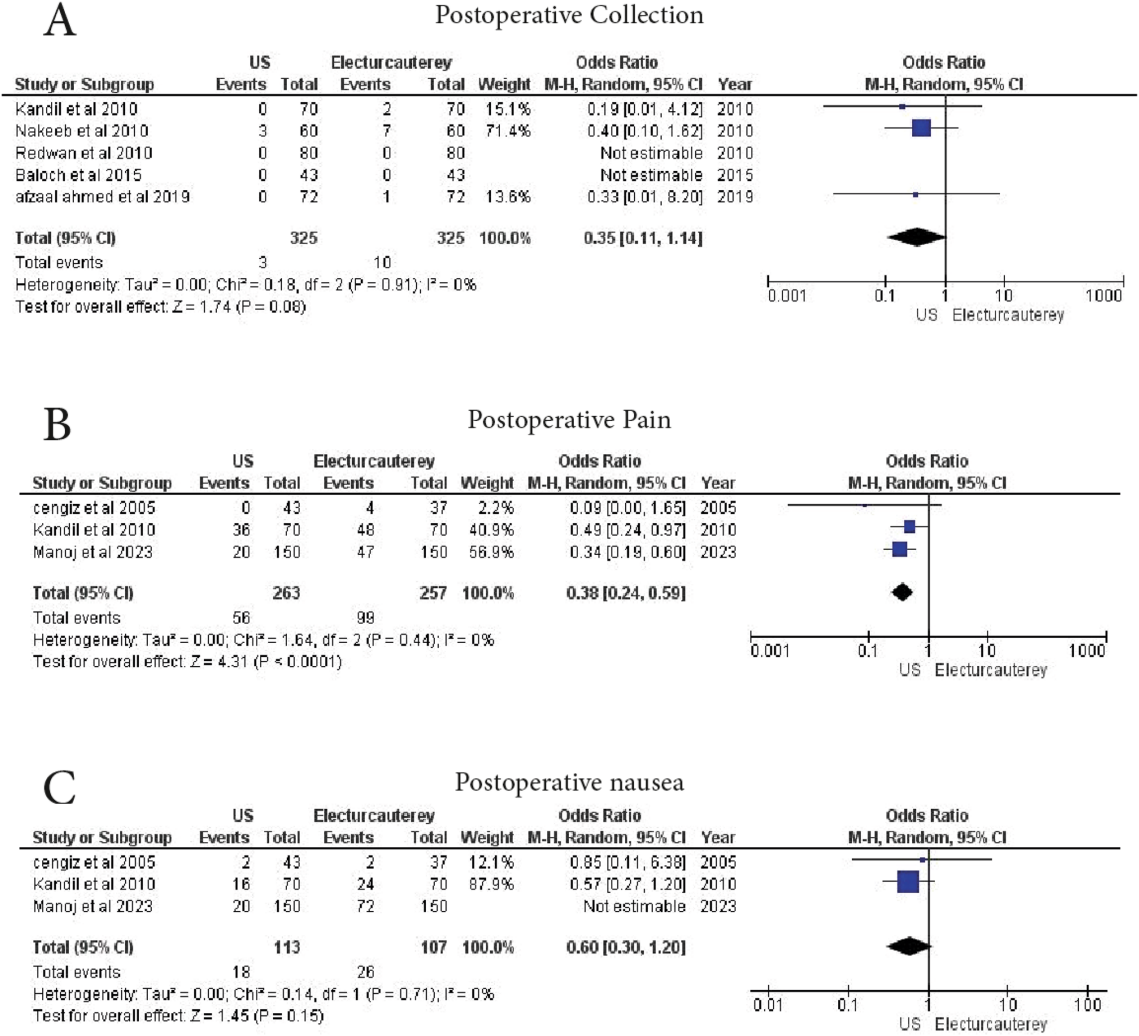
Forest plot show **A** postoperative collection, **B** postoperative pain, **C** postoperative nausea

A total of 5 studies analyzed postoperative collection rates. 325 patients received laparoscopic cholecystectomy under US guidance, and 325 patients received electrocautery. No significant difference was found between the two groups (OR = 0.35, 95% CI 0.11–1.14, *p* = 0.08), with no heterogeneity (I^2^ = 0%) (Fig. 6A)

#### Postoperative pain

A total of 3 studies reported postoperative pain. 263 patients underwent laparoscopic cholecystectomy under US guidance, and 257 patients underwent electrocautery. The US guidance group demonstrated significantly lower postoperative pain (OR = 0.38, 95% CI 0.24–0.59, *p* = 0.0001), with no heterogeneity (I^2^ = 0%) (Fig. 6B)

#### Postoperative nausea

A total of 3 studies analyzed postoperative nausea. 263 patients underwent laparoscopic cholecystectomy under US guidance, and 257 patients underwent electrocautery. No statistically significant difference was found (OR = 0.36, 95% CI 0.13–1.02, *p* = 0.06). However, excluding the study by Manoj et al. (2023), the results remained statistically insignificant (OR = 0.60, 95% CI 0.30–1.20, *p* = 0.15), with no heterogeneity (I^2^ = 0%) (Fig. 6C)

#### Readmission rate

**Fig. 7:**
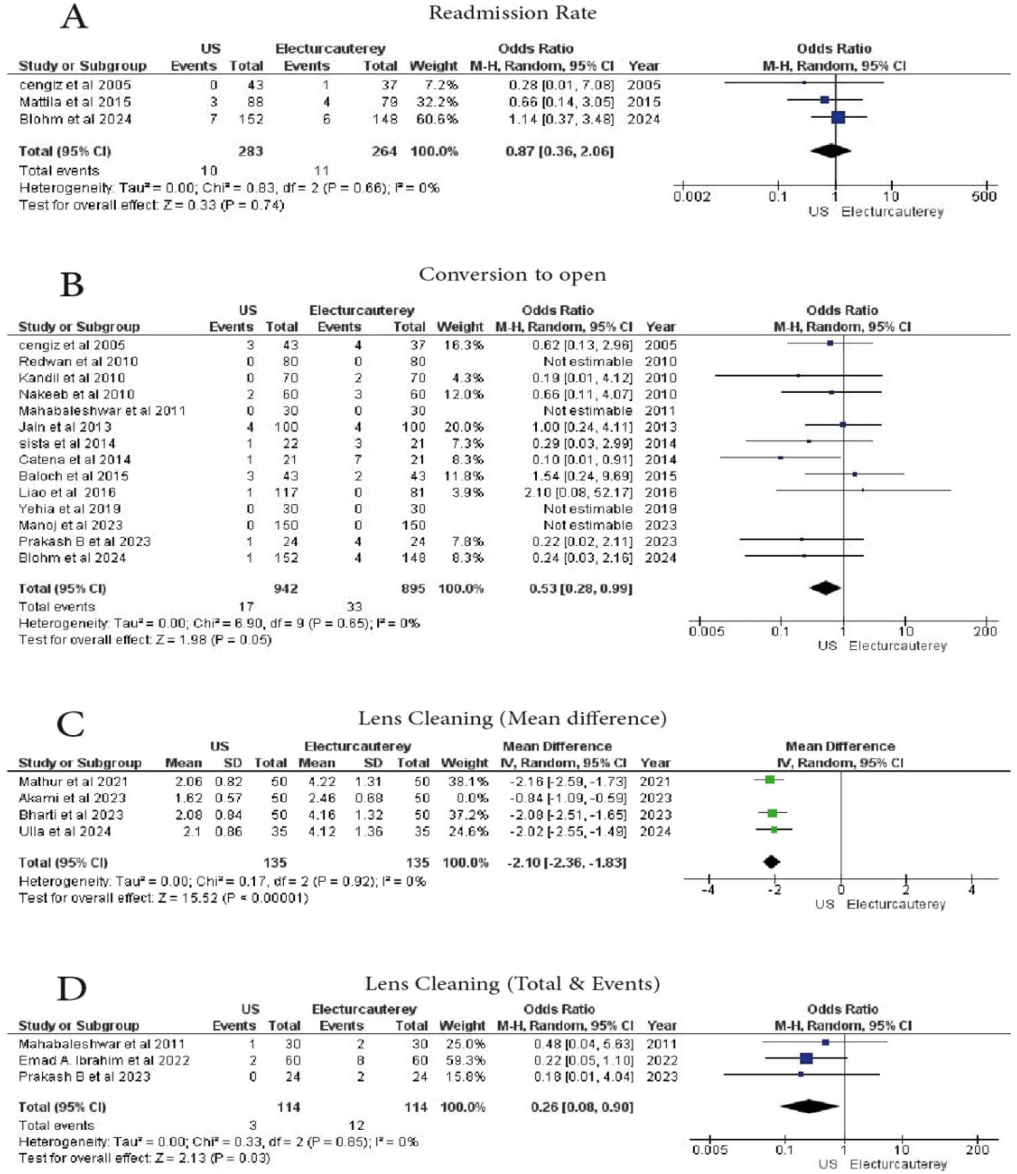
Forest plot show **A** readmission rate, **B** conversion to surgery, **C** Len cleaning (mean difference), **D** Len cleaning (total/event)

3 studies examined the readmission rates. A total of 283 patients underwent laparoscopic cholecystectomy under US guidance, and 264 patients received electrocautery. No statistically significant difference was observed (OR = 0.87, 95% CI 0.36–2.06, *p* = 0.74), with no heterogeneity (I^2^ = 0%) (Fig. 7A)

#### Conversion to open surgery

14 studies reported conversion to open surgery. 895 patients underwent electrocautery, and 942 patients underwent laparoscopic cholecystectomy under US guidance. A statistically significant difference was found (OR = 0.53, 95% CI 0.28–0.99, *p* = 0.05), with no heterogeneity (I^2^ = 0%) (Fig. 7B)

#### Lens cleaning

A meta-analysis of **4 studies** assessed lens cleaning in the two groups. A total of **185 patients** received laparoscopic cholecystectomy under US guidance, and **185 patients** underwent electrocautery. The US guidance group showed a statistically significant reduction in lens cleaning requirements (95% CI − 2.53 to − 0.99, *p* < 0.00001), with substantial heterogeneity (I^2^ = 94%). However, by leaving on out, when excluding the study of Akamai et al. (2023), the findings remained statistically significant (*p* < 0.00001), with no heterogeneity (I^2^ = 0%) (MD = -2.10, 95% CI − 2.36 to − 1.83) (Fig. 7C, D).

## Discussion

Cholecystectomy is a commonly performed operation for patients with symptomatic gallstone disease [37]. Laparoscopic cholecystectomy is currently the standard operation, and it has provided new advancements in gallbladder surgery. It has been proven to be an effective, patient-friendly alternative to open surgery [38, 39].

Many studies have confirmed the efficacy and feasibility of ultrasound (US) since its introduction in LC. At the same time, some reports have already conducted comparative investigations into the use of US over ME, though most of these focused on sealing the cystic artery and tissue division. They also still required conventional clips to ligate the cystic duct. It should be noted that clips have been combined with thermal devices from the start, predominantly being laparoscopic scissors and ME.

Ultrasound is well known as an advanced, minimally invasive surgical instrument for tissue cutting and coagulation. As an alternative to traditional diathermy, US can securely seal vessels up to 5 mm in diameter without clipping the vessels [40]. It also usually provides better management of hemorrhage during dissection in LC than clips combined with laparoscopic scissors and ME, which helps make postoperative bleeding controllable [40].

Ultrasound utilizes the simultaneous functions of cutting, coagulation, and cavitation during tissue dissection by converting electrical energy into high-frequency vibrations (55,500 Hz) [41], referred to as the three primary "C" effects. These high-frequency vibrations generate localized heat between 60 °C and 100 °C, much lower than the temperatures produced during electrosurgery (150 °C) or laser surgery (200 °C) [42]. Consequently, US results in minimal collateral energy transmission to adjacent tissue [43], reducing thermal damage [41] and making tissue separation easier compared to traditional methods involving clips in LC. Notably, the collateral damage from ME, which typically affects the small intestine and common bile duct [43, 44], is rarely encountered when using US [45].

In our study, operative time was shorter in the group treated with laparoscopic cholecystectomy under US guidance (the first group) than in the group treated with electrocautery (the second group). by − 9.13 min. This has many potential advantages, including improving efficiency in the operating room, leads to better financial performance due to enhanced revenue and reduced costs and the Second to room and board costs, operating rooms are the most expensive component of surgical care [46], secondly, An extended operating period increases the risk of infection and exposes incisions to tissue desiccation [47].

Finally, reducing the overall anesthetic time and its complications and increasing the number of cases that can be done on an average operative list, except for Liao et al. 2016 and Shabbir et al. 2016 where operative time was longer in the first group than the second group..

The overall hospital stay in the first group is less than the second group, similar to Abdelhady et al. 2017 study which reported that the harmonic scalpel was associated with shorter operative times, fewer overnight hospital stays, and lower pain scores as a patient's risk of contracting an infection rises with the length of their hospital stay, and an increase in the risk of infection raises length of stay (LOS). Infection specifically results in a 9.3-day increase in LOS. The likelihood of contracting an infection rises by 0.0137 for every day that LOS increases. Even if the chance appears to have increased slightly, it has increased significantly in comparison to the base. (49).

In the present study, the use of laparoscopic cholecystectomy under US guidance was associated with a lower incidence of blood loss and intraoperative complications compared to electrocautery. Additionally, it was associated with a reduced risk of wound infection, postoperative complications, and conversion to open surgery.

The use of laparoscopic cholecystectomy under US guidance was also associated with a lower incidence of gallbladder perforation, stone spillage, and bile leakage compared to the traditional method. Operative time was prolonged in cases of gallbladder perforation in both groups, as stone spillage and bile leakage obstruct the laparoscopic visual field and necessitate frequent instrument exchanges. Notably, lens cleaning was required less often in the first group than in the second.

The first group also had a lower incidence of postoperative pain and nausea according to four studies in our meta-analysis.

In our analysis, we observed minor postoperative collections in both groups, which could be partly attributed to the limited number of patients in each group. Studies also reported comparable readmission rates.

The overall quality of the evidence was moderate for the primary outcome and moderate to high for the further critical outcomes, based on the GRADE criteria. Regarding this, any findings indicating that HEDs are clinically superior to electrocautery for LC should be interpreted cautiously, particularly concerning decreased operating times, length of hospitalisation and blood loss, should be interpreted with caution.

Our meta-analysis faced certain limitations. There were inconsistencies in the studies we included, and we identified a few significant sources of heterogeneity. Moreover, the methods for random sequence generation and follow-up were not clearly defined in some studies. Importantly, although the differences in operative time and blood loss were statistically significant, they lacked clinical relevance. Additionally, our calculation of the number needed to treat suggested that the harmonic scalpel significantly reduces the incidence of gallbladder perforation and postoperative nausea. Another limitation was that we did not evaluate bile duct injuries, chronic pain, or economic outcomes in our study. Consequently, the clinically relevant endpoints included blood loss, postoperative pain, and nausea, as well as gallbladder perforations. With increased clinical effectiveness, the ultrasonic device may outshine electrosurgery alternatives. However, the surgical community has not yet fully embraced this ultrasonic device, even though this study acknowledges the advantages of the harmonic scalpel.

To date, the surgical community has not universally adopted the ultrasonic instrument, though this research recognizes the value of the harmonic scalpel. The cost of the ultrasonic device, limited availability, and the need for retraining in new techniques might be the main challenges. Therefore, we suggest implementing additional training programs for this new technique To assess the effects of laparoscopic cholecystectomy under US guidance on chronic pain, bile duct injuries, or economic outcomes, more research is required in the future.

## Conclusion

In this updated systematic review and meta-analysis, we provided a comprehensive comparison between ultrasonic versus electrocautery dissection in laparoscopic cholecystectomy performed on patients with acute cholecystitis.Based on our analysis, we suggest that ultrasonic dissection has significant advantages over electrocautery with reduced operative time, intraoperative blood loss, faster recovery time and shorter hospital stay. These findings favor ultrasonic dissection as a safer and more efficient technique.Nonetheless, some degree of heterogeneity was observed between the studies included, which might be attributed to differences in study design, differences in surgical technique and clinical characteristics of the patient populations.Long-term patient outcome, cost-effectiveness, and learning curve associated with ultrasonic dissection should be subjects of further research. In general, the selection of surgical technique should be based on patient characteristics and the resources available to achieve optimal clinical outcomes in the treatment of acute cholecystitis.

## Supplementary Information

Below is the link to the electronic supplementary material.

Supplementary file1 (DOCX 726 KB)

## Data Availability

All data generated or analyzed during this study are included in the published article.

## Abbreviations

LC: Laparoscopic cholecystectomy
AC: Acute cholecystitis
UD: Ultrasonic dissection
ED: Electrocautery dissection
RCTs: Randomized controlled trials
WOS: Web of Science
CENTRAL: Cochrane Central Register of Controlled Trials
PRISMA: Preferred Reporting Items for Systematic Reviews and Meta-Analyses
PICO: Population Intervention Comparison Outcomes
BMI: Body Mass Index
RoB 2: Cochrane Risk of Bias 2
RevMan: Review Manager
MD: Mean Difference
CI: Confidence Intervals
OR: Odds Ratios
US: Ultrasound
ME: Monopolar electrocautery
CBD: Common Bile Duct
Pts: Patients
